# The impact of national music activities on improving long-term care for happiness of elderly people

**DOI:** 10.3389/fpsyg.2022.1009811

**Published:** 2022-10-13

**Authors:** Xiaona Zhao, Na Qi, Huizhen Long, Sen Yang

**Affiliations:** ^1^School of Music and the Performing Arts, Mianyang Teachers’ College, Mianyang, China; ^2^School of Philosophy and Sociology, Jilin University, Changchun, China; ^3^Department of Life Culture, Beijing College of Social Administration, Beijing, China; ^4^School of Tourism and Hospitality Management, Hong Kong Polytechnic University, Kowloon, Hong Kong SAR, China; ^5^School of Hotel, Restaurant and Tourism Management, Southern Carolina University, Columbia, SC, United States; ^6^College of Music and Dance, ABA Normal University, A Ba, Sichuan, China

**Keywords:** silver hair, national music, square dancing, body esteem, long-term care and well-being

## Abstract

This paper aims to analyze the influence of national music activities on the long-term care for and happiness of elderly people in the current aging society. Under the popular square dance movement of the whole society, a questionnaire survey was conducted to investigate the differences in the silver-haired body self-esteem and the happiness of the silver-haired exercisers with different exercise methods. Forty first-time square dance participants were selected as experimental objects, and they were divided into an experimental group (ethnic music square dance) and a control group (ordinary music square dance), with 20 people in each group. The results showed that the effective recovery rate of the questionnaire was 95.10% (136/150). There were 47, 45, and 44 people in the square dance, other sports, and non-sport groups, respectively. The total scores of physical self-esteem, physical self-worth, physical quality, health worry, satisfaction and interest in life, control of emotion and behavior, and happiness of silver-haired people who participated in square dance activities were higher than those of other sports players (*p* < 0.05). The total scores of physical self-esteem, exercise ability, physical condition, and physical quality scores were significantly higher than those of non-sports people (*p* < 0.01), and each factor and total score on the happiness were higher than those of non-sports people (*p* < 0.05). The body weight, waist circumference, hip circumference, and thigh circumference of the experimental group after exercise were significantly different from those before exercise (*p* < 0.01), and the factors of the body self-esteem scale and well-being scale were higher than those before the experiment (*p* < 0.05). This shows that music has a positive effect on the long-term care for and happiness improvement of the silver-haired family, which can improve the physical and mental health of the silver-haired family and further improve the quality of life of the silver-haired family in their later years. This offers a theoretical basis for the development of the elderly folk music square dance movement in the future and provides a reference for the formulation of silver-haired patriarchal photos and happiness intervention programs.

## Introduction

With the rapid development of today’s society, the aging of the population is accelerating, and China is entering an “aging” society (Lei, 2018; Siyal et al., 2021). According to statistics, by the end of 2020, there were approximately 264 million elderly people aged 60 and above in China, accounting for approximately 18.7% of the total population (McLaughlin et al., 2020). As an important part of the family structure, the improvement of the health and happiness of the silver-haired people is of great significance to the construction of a harmonious society and the cornerstone of social development and progress (Yang et al., 2017; Ota et al., 2018). An increase in age will lead to a decline in various physical functions of the elderly to varying degrees, body immunity and resistance will also decline, and the corresponding diseases will increase, resulting in the reduction or suspension of physical activities. Long-term non-participation in physical activities cannot stimulate the corresponding functions of the body, accelerate the decline of body functions, and form a vicious circle, which leads to a decrease in the quality of life of the elderly (Eckstrom et al., 2020; Weyh et al., 2020). Faced with the phenomenon of “aging,” what should we do is to improve the quality of life of the elderly (Ruggieri et al., 2019)?

Currently, music activities play an important role in the lives of silver-haired people (Wattanasoei et al., 2017). Relevant studies have shown that music has a positive impact on the mental and physical health of silver-haired people (Vinciguerra, 2017). Therefore, it is a major direction of music research to improve the physical and mental health of silver-haired people by making them contact with music (Sampaio et al., 2017). However, with increasing age, the physiological function and physical quality of the silver-haired group gradually degenerated, and most of the silver-haired group did not want to move. As a public place, square dance aims at entertainment and exercise and combines a large number of music songs, which are popular with the majority of silver-haired people (Petrini, 2019). Compared with other dance forms, square dance has moderate movement intensity, a strong sense of rhythm, an active atmosphere, and a cheerful style, which brings silver-haired people a sense of psychological pleasure and is especially suitable for silver-haired people to participate in (Li et al., 2018). In addition, this movement can be implemented even without a dance foundation, which is widely accepted and can bring sufficient long-term care and happiness to the elderly (Stevenson, 2017). China is a multi-ethnic country, and each ethnic group has its unique national music and dance. Different national music dances have different expression techniques in movement, music, emotion, and other aspects (Cave, 2017; Fauteux, 2017; Sebastian, 2018). Whether the elements of folk music and dance integrated into modern square dance will have an impact on the silver-haired patriarch’s photo and happiness is unknown.

National music and square dance were integrated by relying on the popular square dance movement of the whole society. Through a literature review, questionnaire survey, and experiment, the influence of national music square dance on the body characteristics, body self-esteem, and happiness of silver nationality was explored to improve the quality of life of silver nationality and provide a scientific basis for carrying out elderly national music square dance in the future.

## Literature review

### Analysis of the influence of square dance on happiness

The square dance itself has a positive role in strengthening the body, entertaining the body and mind, and entertaining the public (Yu et al., 2020). Exercising through square dancing can improve the physical health of individuals, regulate psychology (Xie et al., 2021), and positively affect the quality of life of elderly individuals. The Square dance movement is an artistic expression of dancing in public places, according to the accompaniment of music. Square exercise can improve an individual’s physical health and psychological adjustment. Liu and Guo (2013) mund in their study that square dancing could improve the happiness of middle-aged and elderly women, and their happiness index was significantly higher than that of other people who did not participate in square dancing. Wang (2015) combined northeast Bangko dance with a square dance and found that dance could improve lung capacity, physical and mental health, and quality of life without the requirements of clothes, equipment, and stage. It was widely accepted and promoted the happiness of the elderly Xu and Mao (2013).

### Analysis of the influence of music on happiness

National music is a common form of artistic expression, and its positive influence on individuals exists in all aspects. Du et al. (2017) studied the effect of music therapy on the happiness of border guards and found that by rendering the environment of music for officers and soldiers in sanitaria, the level of physical self-esteem of officers and soldiers was significantly improved, and their happiness was also increased. Xu and Mao (2013) combined jogging with music to study its influence on the improvement of college students’ happiness. The results showed that jogging and jogging combined with music had significant differences in the happiness of college students, and jogging combined with music could eliminate the psychological fatigue of college students (Xu and Mao, 2013). At present, a large number of studies have pointed out that music can effectively relieve the negative emotions of elderly individuals (Khazaee-Pool et al., 2015; Diego et al., 2018). The researchers of Zahmatkesh et al. (2018) pointed out that music can improve the spiritual development of the elderly and effectively increase the happiness index and degree of life of elderly individuals. Some researchers have pointed out that group music activities can improve the subjective well-being and leisure satisfaction of elderly people (Bonato et al., 2020). The results of Lee and Lee (2020) pointed out that at the psychological level, music can bring happiness to the elderly and reduce or eliminate pain; at the emotional level, music brings comfort or beauty to the elderly and reduces or eliminates discomfort or ugliness; and at the soul level, music can bring happiness to elderly individuals, sublime or transcend suffering.

### The research status of happiness of silver-haired people

The silver hair nationality is an important part of the family structure. The health and happiness of the silver hair nationality are of great significance to the construction of a harmonious society and are the cornerstone of the realization of social development and progress. Yang et al. (2021) analyzed the influence of the family and health status of the elderly on happiness through a questionnaire survey and found that harmonious family relationships, healthy family communication, and the health status of the elderly all have a positive impact on happiness. Chen (2017) studied the influence of the family environment on the improvement of happiness in elderly people and found that elderly people with a good family environment had higher subjective happiness, which was positively correlated with the intimacy and emotional expression of the family environment. Cao et al. (2015) discussed the influencing factors of the community elderly’s happiness index and found that the elderly’s happiness index was positively correlated with their health status, stress response, and future goals, suggesting that relevant departments should formulate relevant policies to improve elderly’s happiness.

According to the above research, both square dancing and music have positive effects on the improvement of happiness, and the improvement of happiness of silver-haired people is influenced by various environments. With the continuous development of square dance, its music forms are also diverse, but the application of national music in square dance is rarely reported, and the integration of the two to improve the long-term care and happiness of silver-haired people is not much involved. Under the above situation, national music and square dance were integrated, and the influence of national music and square dance on the long-term care and happiness of silver-haired people was explored through a literature review, questionnaire survey, experiment, and other methods.

## The fusion of national music and square dance

### The application of national music in square dance

China has 56 ethnic groups, each of which has its unique national music and dance, which contains People’s Daily life and cultural deposits. Different ethnic music dances have different expression techniques in movement, music, and emotion, which are gradually absorbed by modern square dance. To integrate the elements of national music into the square dance, it is necessary to adapt the square dance according to the music style of various nationalities, improve the quality of square dance choreographers, and innovate the expression form of national music square dance (Kim et al., 2010).

Square dance is not significantly different from other dances. It is an art combining dance with music, and dancers create appropriate dance movements according to the type and rhythm characteristics of musical accompaniment. National music was added to the creation of square dance. At the same time, as hearing the national music, dance performance will have a soul. This requires choreographers to improve their music literacy, grasp the style characteristics of various ethnic music and analyze them to seek common ground with square dancing to better integrate ethnic music into square dancing.

Choreographers are the key to the charm of square dancing, who are required to have a deep music dance foundation. To improve choreographers’ music quality and integrate ethnic music, choreographers must have close contact with ethnic minorities, understand their customs and culture, feel their music and dance connotations, and have in-depth communication. Only in this way can a square dance with national flavor be created.

The fusion and adaptation of square dance and national music often depend on the dancers’ conditions. If the dancers are graceful and soft, Xinjiang elements can be added accordingly. If the character of the dancers is bold and forthright, Mongolian elements can be added. For dancers living in the northwest, elements such as Bangko dance, gong, and drum can be added. For the silver-haired group in our study, tai chi, fans, and other classical elements can be added to it. Only according to the characteristics of national music, combined with the corresponding elements, square dance can have real charm (Fu et al., 2017).

### Related research methods

Firstly, the literature material method was used. Through consulting and sorting out relevant papers, periodicals, newspapers, and other literature, the influence of the national music square dance on the mental mood, long-term care, and happiness of the silver-haired people was analyzed. These documents provide a theoretical reference for the study of this research.

The subjects of this questionnaire survey are 50 randomly selected silver-haired exercisers who participated in square dance activities, other sports, and non-sports activities in Xi’an, totaling 150. Questionnaires were distributed to exercisers from 7: 00 to 9: 00 p.m. and collected on the spot. The “Physical Self-Esteem Scale (PSPP)” and “Well-Being Scale (GWB)” is used to test the happiness of the respondents. The content of the questionnaire includes three parts: basic information of subjects, body self-esteem scale, and happiness scale. The basic information of the subjects mainly includes information such as age, gender, height, weight, exercise mode, exercise time, weekly exercise times, and the duration of each exercise. First, 50 respondents who participated in square dancing activities were distributed the exercise form of silver nationality square dancing. According to the results, the criteria of the results were determined. The exercise standards are shown in Table 1. Exercise time is required to be more than 1 year, no less than 3 times a week, and no less than 30 min each time (Qian-li, 2018). The physical self-esteem scale (PSPP) and well-being scale (GWB) were distributed and recycled to those who met the exercise standards. The questionnaire will not be issued to those who do not meet the standards.

**Table 1:** Silver hair square dance exercise standard.

Exercise time (year)	Number of workouts (times/week)	Exercise time (min/time)
≥ 1	≥ 3	≥ 30

PSPP is one of the main tools used to measure mental health indicators (Carmel, 2017; Mignani et al., 2017; Tsay et al., 2018). Marsh et al. proposed a simple structural model for the first time in 1988, and Fox et al. established a standardized and personalized multidimensional hierarchical model based on which Xu Xia further improved in 2001. The body self-esteem scale is a measure of general body self-worth and subdomains, which have high credibility (Steffl et al., 2017; Wimo et al., 2017). All the questions in the scale have two statements. The investigator chose “completely consistent” or “somewhat consistent” according to the actual situation. There were 30 questions, each with a score ranging from 1 to 4. The higher the score, the higher the self-esteem.

The general well-being scale (GWB) is a standard measure developed by the national center for health statistics to evaluate participants’ well-being statements (Yang et al., 2017; Delhom et al., 2018). There were a total of 18 test items; the higher the score was, the higher the happiness. The correlation between the score of individual items and the total score of the scale is between 0.48 and 0.83, which indicates good reliability and validity.

Thirdly, experimentation: 40 silver-haired people who have never participated in square dancing in Xi’an city were randomly selected as subjects. Among them, 20 were male and 20 were female, aged between 60 and 65, and there were no serious diseases. The 40 subjects were divided into the experimental group and the control group, as shown in Table 2. The experimental group was the ethnic music square dance exercise group, which only participated in the exercise of ethnic music square dance. In the control group, there were 20 people in each group and 10 men and 10 women in each group.

**Table 2:** Grouping of subjects.

Group	Male	Female	Average age
Experimental group	10	10	61.5
Control group	10	10	62.3

The experimental group participated in the square dancing exercise for no less than 1 h three times a week for 2 months. The exercise time of the control group was the same as that of the experimental group. Before and after the experiment, 40 subjects were given the body self-esteem scale (PSPP) and the well-being scale (GWB). After filling in, the subjects’ waist circumference, hip circumference, calf circumference, forearm circumference, and other physical characteristics were measured.

Fourthly, mathematical statistics are employed. The collected data were sorted out, SPSS 20.0 software was used to analyze the data results, and a *t*-test of samples was conducted.

## The influence of the national music square dance on the long-term care and happiness of the silver-haired people

### Questionnaire results

Table 3 shows the distribution and recovery results of this questionnaire. A total of 150 questionnaires were distributed, 143 of which were recovered. A total of 136 questionnaires were valid, including 47 for square dancing, 45 for running and other sports, and 44 for non-sports.

**Table 3:** The results of the distribution and recovery of the questionnaire.

Total number of issued	Number of recycling	Recovery	Valid number of questionnaires	Square dancing	Other sports	Nonphysical activity
150	143	95.3%	136	47	45	44

Table 4 shows the square dance exercise of the silver-haired people who participated in the questionnaire. The exercise time of all the survey subjects was more than 1 year, most of them exercised more than 3 times, and most of them exercised for more than 30 min each time.

**Table 4:** Questionnaire about the square dance exercise of the silver hair nationality.

Basic condition	Exercise time (year)				Number of workouts (times/week)				Exercise time (min/time)		
	0.5–1	1–2	2–3	>3	1–2	3–4	5–6	>6	<30	30–60	>60
Number	0	20	15	12	4	25	10	8	6	32	9

The investigators who participated in square dancing, other sports, and non-sports were tested with the body self-esteem scale, and the sample *t*-test was conducted. The results are shown in Table 5.

**Table 5:** Participants in square dancing, other sports, non-sports, the silver hair people’s body self-esteem scale.

Factor	Square dance movement	Other sports	t	p	Nonphysical activity	t	p
Body self-worth	9.017 ± 1.426	8.532 ± 1.363	0.816	0.032*	8.517 ± 1.353	1.098	0.012*
Athletic ability	9.097 ± 1.543	8.986 ± 1.170	0.827	0.068	8.556 ± 1.370	0.658	0.003**
Physical condition	9.108 ± 1.746	9.002 ± 1.353	0.512	0.070	8.342 ± 1.453	0.626	0.006**
Physical attraction	9.220 ± 1.744	8.993 ± 1.658	0.748	0.067	8.363 ± 1.758	0.644	0.012*
Physical quality	9.496 ± 2.268	9.113 ± 1.397	0.797	0.036*	8.583 ± 1.467	0.646	0.009**
Total score	47.167 ± 7.425	45.336 ± 4.713	−0.776	0.017	40.373 ± 4.843	0.696	0.003**

^*^Indicates a significant difference compared with the square dancers, *p* < 0.05. ^**^Indicates a significant difference compared with the square dancers, *p* < 0.01.

According to the data in Table 5, by comparing the test results of the body self-esteem scale of square dancing and other sports, it can be concluded that the scores of various data of square dancing are higher than those of other sports, especially in the two factors of body self-worth and physical fitness. Moreover, the *p*-values of the two factors were less than 0.05 in the statistical test, showing a significant difference. By comparing the test results of the body self-esteem scale of square dancing and non-physical exercise, it was concluded that the scores of all data for square dancing were higher than those of non-physical exercise, and the *p*-values of statistical tests were all less than 0.05, showing a large difference. It can be concluded that those with silver hair who participate in square dancing have the highest body self-esteem. This is because the silver-haired people who participate in the square dance exercise are more confident about their physical conditions and physical fitness, believing that they have a higher level of physical health, can participate in various sports, and thus have higher body self-esteem.

Table 6 shows the happiness scale of silver-haired people participating in square dancing, other sports, and non-sports. It showed that in addition to the two factors of melancholy or happy mood and anxiety, the other happiness factors of the silver-haired people who participate in the square dance were all higher than those of the silver-haired people who participate in other sports. Moreover, the *p*-values of the three factors of health concern, life satisfaction, and interest, and emotional and behavioral control were all less than 0.05, indicating that there was a great difference among the three factors. The happiness level of the silver-haired people who participated in square dancing was slightly higher than that of the silver-haired people who participated in other sports. However, happiness factors participating in square dancing were all higher than those in non-sports, and the *p*-values in statistical tests were all less than 0.05, showing a significant difference, indicating that the happiness level of silver-haired people participating in square dancing was much higher than that of silver-haired people participating in non-sports. This is because the gray-haired people who take part in square dancing can better regulate their mood. Although they sometimes worry about their health, they will relax most of the time and thus enjoy the highest happiness (Liu and Guo, 2013; Qian and Lu, 2019). In terms of happiness, people can improve their positive sense of honor and mutual intimacy during exercise, thereby enhancing their subjective happiness. Research results show that not all forms of exercise are conducive to the improvement of subjective happiness, and high-intensity, long-duration, and relatively single exercise cannot produce positive emotions. Under normal circumstances, gentle aerobic exercise is more likely to make people have a positive experience. As regular square dance practice, it can not only relieve negative emotions such as depression and anxiety but also generate positive emotions (Carmel, 2017). During the square dance exercise, the mind and body are combined, the brain follows the rhythm, the body makes standardized movements, and the whole body is devoted to it, which can eliminate adverse interference and maintain a high degree of consistency between the heart and the behavior (Steffl et al., 2017). Square dance exercises can improve the subjective happiness of elderly people by enhancing positive emotions and improving cognition (Delhom et al., 2018). During the square dance exercise, the exercisers achieve psychological satisfaction through the control of their own bodies, and through communication, enhance their sense of identity, enhance their self-confidence, increase their positive emotions and life satisfaction, and thus improve their subjective well-being (Yang et al., 2017).

**Table 6:** Participants in square dancing, other sports, and non-sports on the well-being scale of silver hair.

Factor	Square dance movement	Other sports	*t*	*p*	Nonphysical activity	*t*	*p*
Worries about health	7.68 ± 0.93	7.42 ± 0.93	0.391	0.027*	7.24 ± 1.21	0.960	0.021*
Vigor	13.85 ± 1.54	13.66 ± 1.48	1.568	0.086	13.44 ± 1.54	0.333	0.024*
Satisfaction and interest in life	8.79 ± 1.33	8.36 ± 1.35	2.613	0.013*	7.38 ± 1.56	3.352	0.010*
A state of melancholy or cheerfulness	11.32 ± 1.76	11.42 ± 1.61	−0.107	0.937	10.94 ± 2.04	1.568	0.030*
Control of emotions and behavior	14.72 ± 1.84	14.32 ± 1.99	1.682	0.035*	13.36 ± 2.13	0.941	0.045*
Anxiety	17.30 ± 2.24	17.62 ± 2.32	−1.252	0.809	15.54 ± 2.56	0.415	0.018*
Total score	66.47 ± 7.53	66.22 ± 7.48	1.041	0.029*	62.03 ± 9.02	3.258	0.025*

^*^Indicates a significant difference compared with square dancers, *p* < 0.05.

### Experimental results

Before the beginning of the experiment, the physical characteristics of the silver-haired people in the experimental group and the control group were measured and averaged. The results are shown in Table 7. According to the data in the table, the statistical *p*-values of the physical characteristics of the experimental group and the control group were all >0.05, with no significant difference, indicating that the body shapes of the experimental group and the control group were the same.

**Table 7:** Physical characteristics of the silver-haired people in the experimental group and the control group before the experiment.

Factor	Experimental group	Control group	*t*	*p*
Height	159.83	160.83	2.96	0.399
Weight	60.63	59.00	0.36	1.149
Waistline	82.79	79.25	1.68	0.731
Hip line	96.17	94.40	0.42	0.661
Thigh circumference	54.33	51.12	1.72	0.922
Calf circumference	34.66	34.88	1.81	0.621
Forearm circumference	24.83	24.98	1.88	0.827

Table 8 shows the mean body self-esteem scores of the experimental group and the control group before the experiment. It showed that before the experiment, the scores of the body self-esteem factor of the silver hair group in the experimental group and the control group are close, and the statistical value of p was mostly greater than 0.05, with little difference, indicating that the body self-esteem of the experimental group and the control group were at the same level before the experiment.

**Table 8:** The body esteem scale of the experimental group and the control group before the experiment.

Factor	Experimental group	Control group	*t*	*p*
Body self-worth	9.250	8.617	1.403	0.370
Athletic ability	9.217	9.717	−0.796	0.503
Physical condition	9.417	9.550	−0.092	0.965
Physical attraction	9.383	9.517	−0.080	0.974
Physical quality	9.617	9.750	−0.075	0.978
Total score	46.317	46.550	0.047	1.074

Table 9 shows the happiness scores of the experimental group and the control group before the experiment. The score of the happiness factor of the silver-haired people in the experimental group and the control group was the same, and the statistical *p*-values were all greater than 0.05, showing no significant difference, indicating that the happiness of the experimental group and the control group were at the same level before the experiment.

**Table 9:** The well-being scale of the experimental group and the control group before the experiment.

Factor	Experimental group	Control group	*t*	*p*
Worries about health	6.18	6.25	−0.086	0.970
Vigor	12.42	12.52	−0.112	0.949
Satisfaction and interest in life	5.15	5.35	−0.561	0.663
A state of melancholy or cheerfulness	9.92	9.95	0.081	1.100
Control of emotions and behavior	9.62	9.52	0.375	0.978
Anxiety	13.18	13.15	0.211	1.107
Total score	55.72	55.98	0.029	1.059

After a 2-month exercise of national music square dance in the experimental group, the physical characteristics of the experimental group and the control group under the same exercise conditions are shown in Table 10. After comparing the physical characteristics of the silver-haired people in the experimental group and the control group, it was concluded that the height, calf circumference, and forearm circumference of the two groups were not significantly different, while the weight of the experimental group was lower than that of the control group, showing a significant difference. At the same time, because the national music is relatively more straightforward than the ordinary music, the action range was larger, which made the waist circumference, hip circumference, and thigh circumference of the silver hair group that participates in the national music square dance lower than that of the silver hair group that participates in the ordinary music square dance.

**Table 10:** After the experiment, the physical characteristics of the silver-haired people in the experimental group and the control group were compared.

Factor	Experimental group	Control group	*t*	*p*
Height	160.63	160.83	0.89	0.512
Weight	58.71	58.99	5.32	0.162
Waistline	80.79	79.25	4.58	0.159
Hip line	94.93	94.40	3.29	0.180
Thigh circumference	52.73	51.12	7.84	0.163
Calf circumference	34.84	34.88	1.10	0.733
Forearm circumference	24.25	24.32	1.86	0.852

Table 11 shows the body self-esteem scores of the experimental group and the control group after the experiment. All the factors of body self-esteem in the experimental group were higher than those in the control group, and all the *p*-values were less than 0.05, showing a significant difference. The body self-esteem of the experimental group was significantly higher than that of the control group, indicating that the body self-esteem of the participants in the national music square dance was significantly higher than that of the participants in the ordinary music square dance.

**Table 11:** Body esteem scale of the experimental group and the control group after the experiment.

Factor	Experimental group	Control group	*t*	*p*
Body self-esteem	18.25	16.25	2.066	0.021
Athletic ability	17.42	15.75	2.510	0.032
Physical condition	18.05	16.02	3.160	0.012
Physical attraction	17.71	15.92	1.851	0.007
Physical quality	17.61	16.35	0.702	0.009
Total score	88.68	79.68	2.286	0.01

Table 12 shows the happiness scores of the experimental group and the control group after the experiment. All factors of happiness in the experimental group were higher than those in the control group, and the statistical *p*-values were all less than 0.05, showing a significant difference. The happiness in the experimental group was significantly higher than that in the control group, indicating that participating in the national music square dance could obtain more happiness.

**Table 12:** The well-being scale of the experimental group and the control group after the experiment.

Factor	Experimental group	Control group	*t*	*p*
Worries about health	6.45	6.25	−0.645	0.185
Vigor	12.58	12.52	−0.028	0.181
Satisfaction and interest in life	7.32	6.25	3.347	0.157
A state of melancholy or cheerfulness	10.32	9.95	−0.658	0.577
Control of emotions and behavior	13.22	12.32	2.018	0.202
Anxiety	16.82	14.58	2.129	0.155
Total score	65.95	61.12	2.422	0.182

Through intragroup comparisons of body self-esteem and happiness scores before and after the experiment in the experimental group and the control group, the influence of national music square dance on the well-being of people with silver hair was further explored.

**Figure 1:**
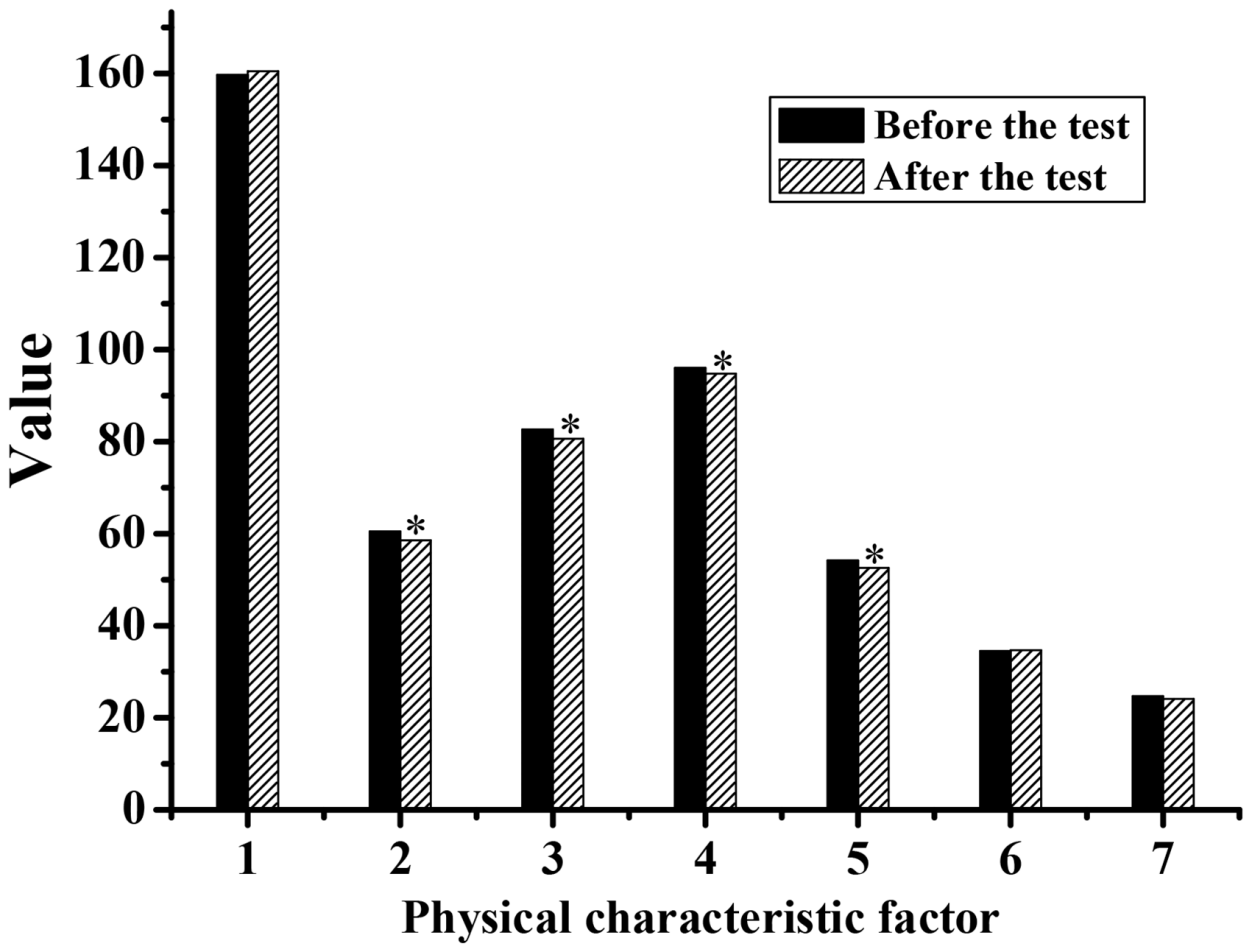
Comparison of the physical characteristics index values of the silver-haired people in the experimental group before and after the experiment. (1: height; 2: weight; 3: waist circumference; 4: hip circumference; 5: thigh circumference; 6: calf circumference; 7: forearm circumference; * indicates a significant difference compared with before the test, *p* < 0.05).

**Figure 2:**
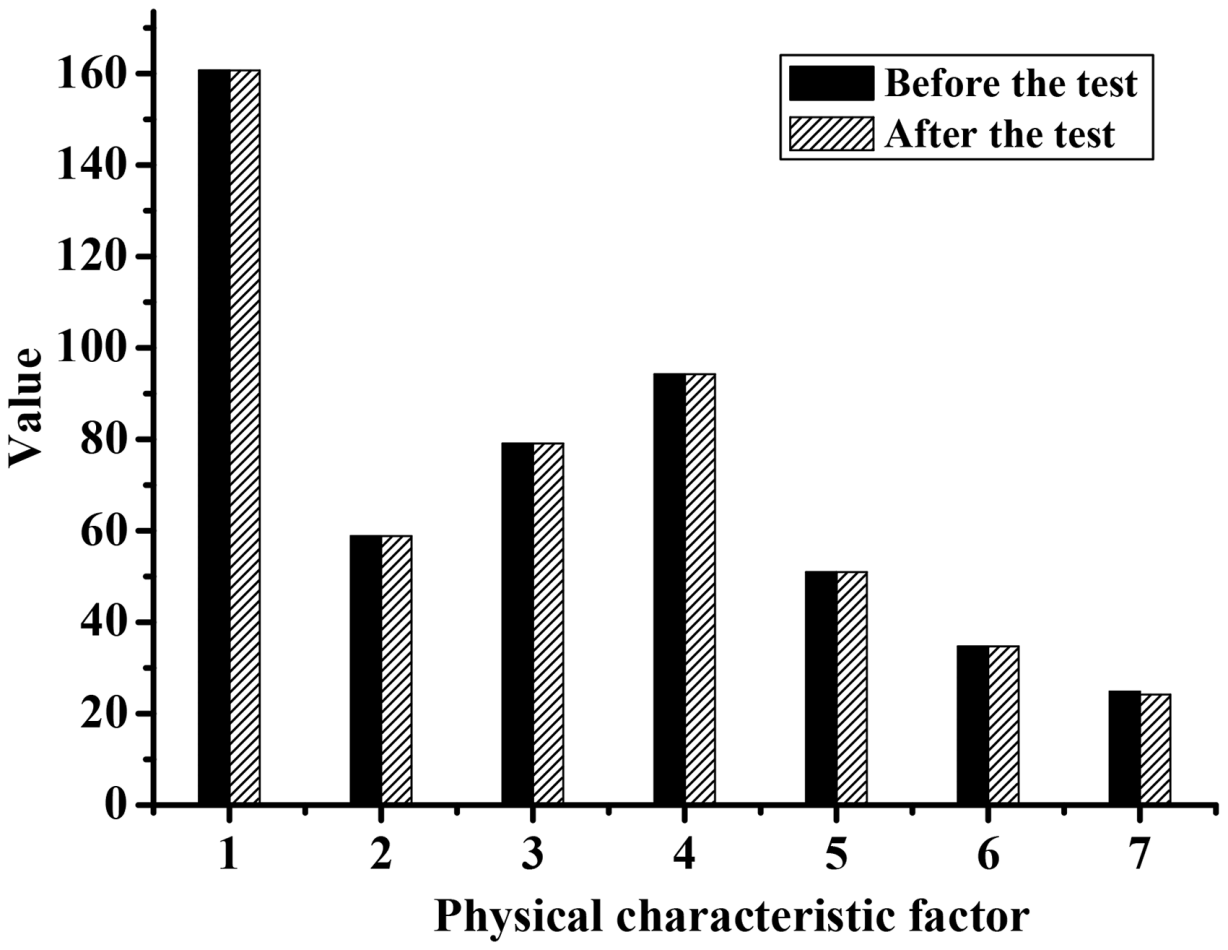
Comparison of physical characteristics index values of silver-haired people in the control group before and after the experiment. (1: height; 2: weight; 3: waist circumference; 4: hip circumference; 5: thigh circumference; 6: calf circumference; 7: forearm circumference).

Figures 1, 2 show the physical characteristics of the experimental group and the control group before and after the experiment, respectively. It can be concluded that the body weight, waist circumference, hip circumference, and thigh circumference measurements were all significantly lower than those before exercise (*p* < 0.05). This was because the characteristics of the national music combined with the square dance movement can make the silver hair people’s weight reduction, waistline reduction, hip curve more beautiful, and thighs also became more solid. However, the statistical *p*-value of the control group was mostly greater than 0.05, with no significant difference.

**Figure 3:**
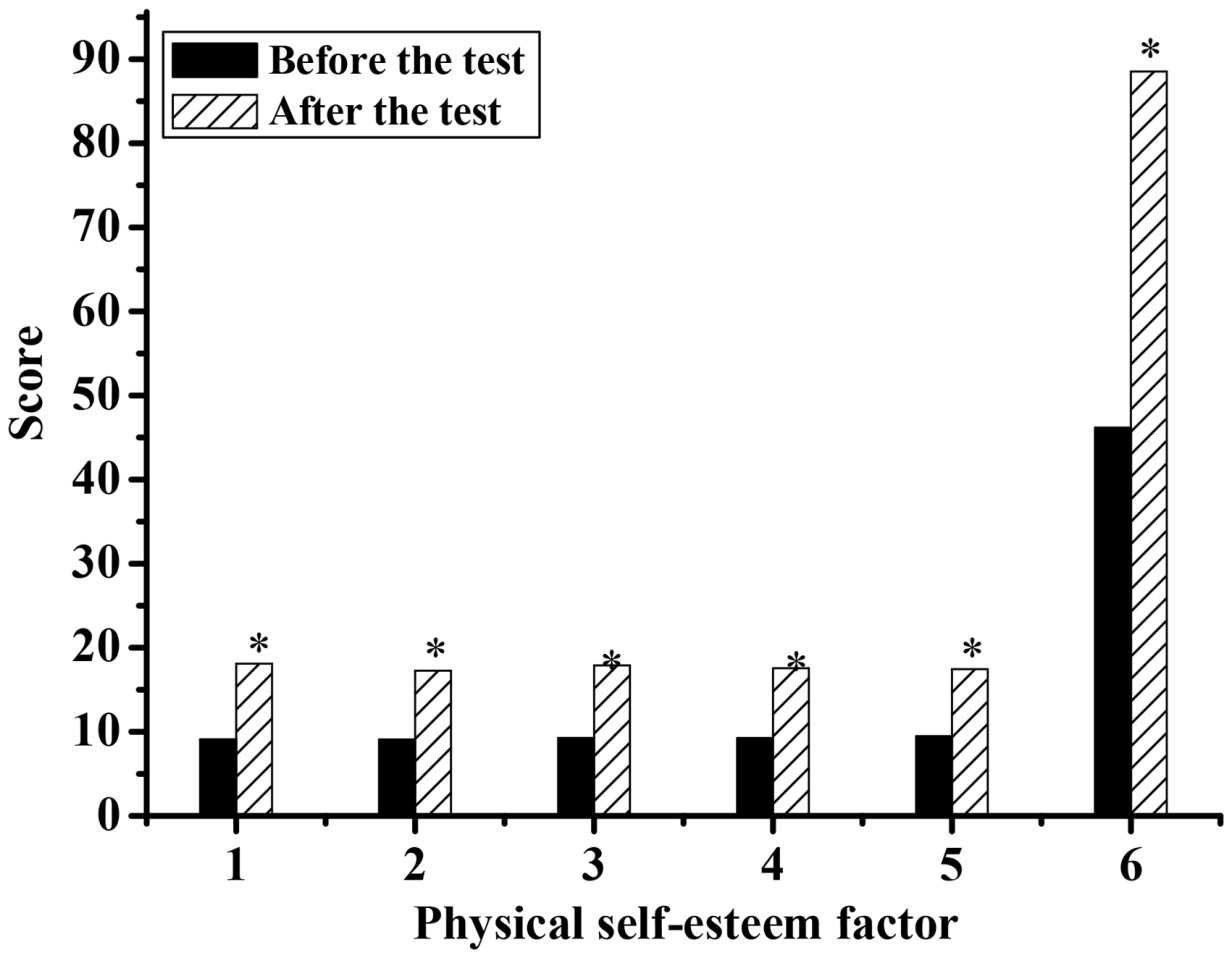
Comparison of physical self-esteem scale scores of silver-haired people in the experimental group before and after the experiment. (1: physical self-worth; 2: athletic ability; 3: physical conditions; 4: physical attraction; 5: physical fitness; 6: total score; * indicates a significant difference compared with before the test, *p* < 0.05).

**Figure 4:**
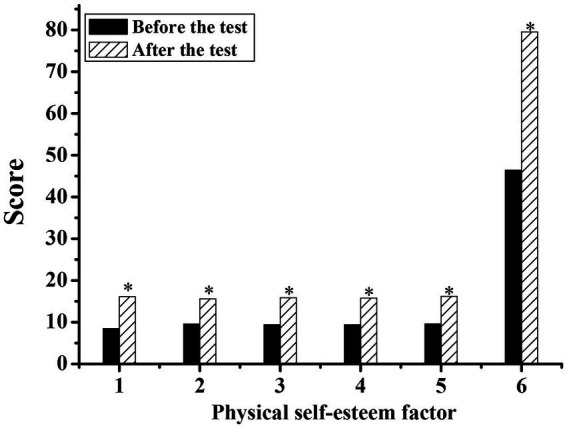
Comparison of physical self-esteem scale scores of silver-haired people in the control group before and after the experiment. (1: physical self-worth; 2: athletic ability; 3: physical conditions; 4: physical attraction; 5: physical fitness; 6: total score; * indicates a significant difference compared with before the test, *p* < 0.05).

Figures 3, 4 show the body self-esteem scores of the experimental group and the control group before and after the experiment, respectively. By comparing the relevant data before and after the experiment, it was concluded that all the body self-esteem factors in the experimental group were higher than those before the experiment, and the statistical *p*-values of all factors were less than 0.05, showing a significant difference, indicating that national music had improved the body self-esteem level of the silver group to a certain extent. The body self-esteem factor of the control group after the experiment was slightly higher than that before the experiment, and the statistical *p*-value was less than 0.05, showing a significant difference. The body self-esteem level after the experiment also increased, but the degree of improvement was smaller than that of the experimental group.

**Figure 5:**
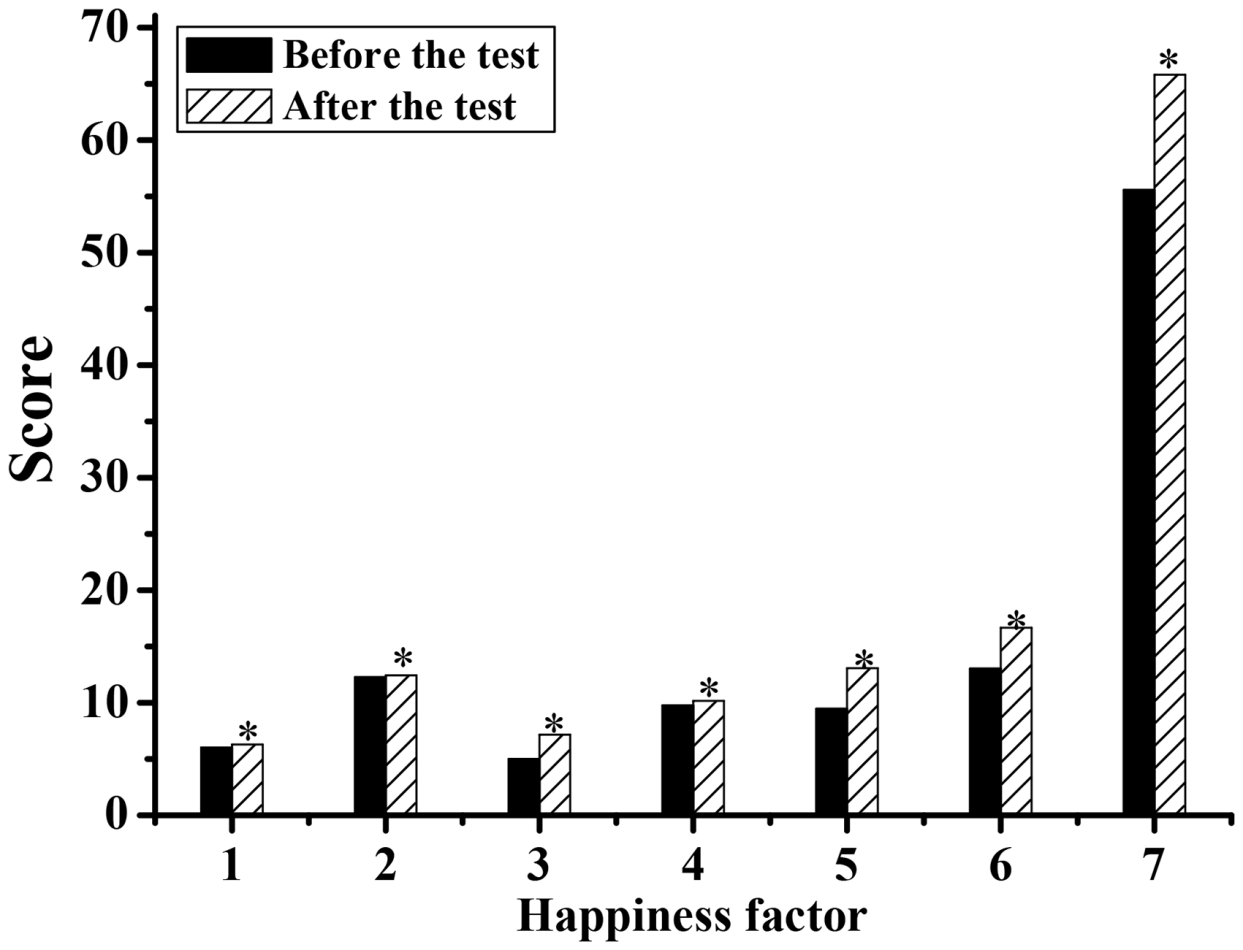
Comparison of the scores of the silver-haired happiness scale in the experimental group before and after the experiment. (1: worry about health; 2: energy; 3: satisfaction and interest in life; 4: depressed or happy mood; 5: control of emotion and behavior; 6: anxiety; 7: total score; * suggests there is a significant difference compared to before the test, *p* < 0.05).

**Figure 6:**
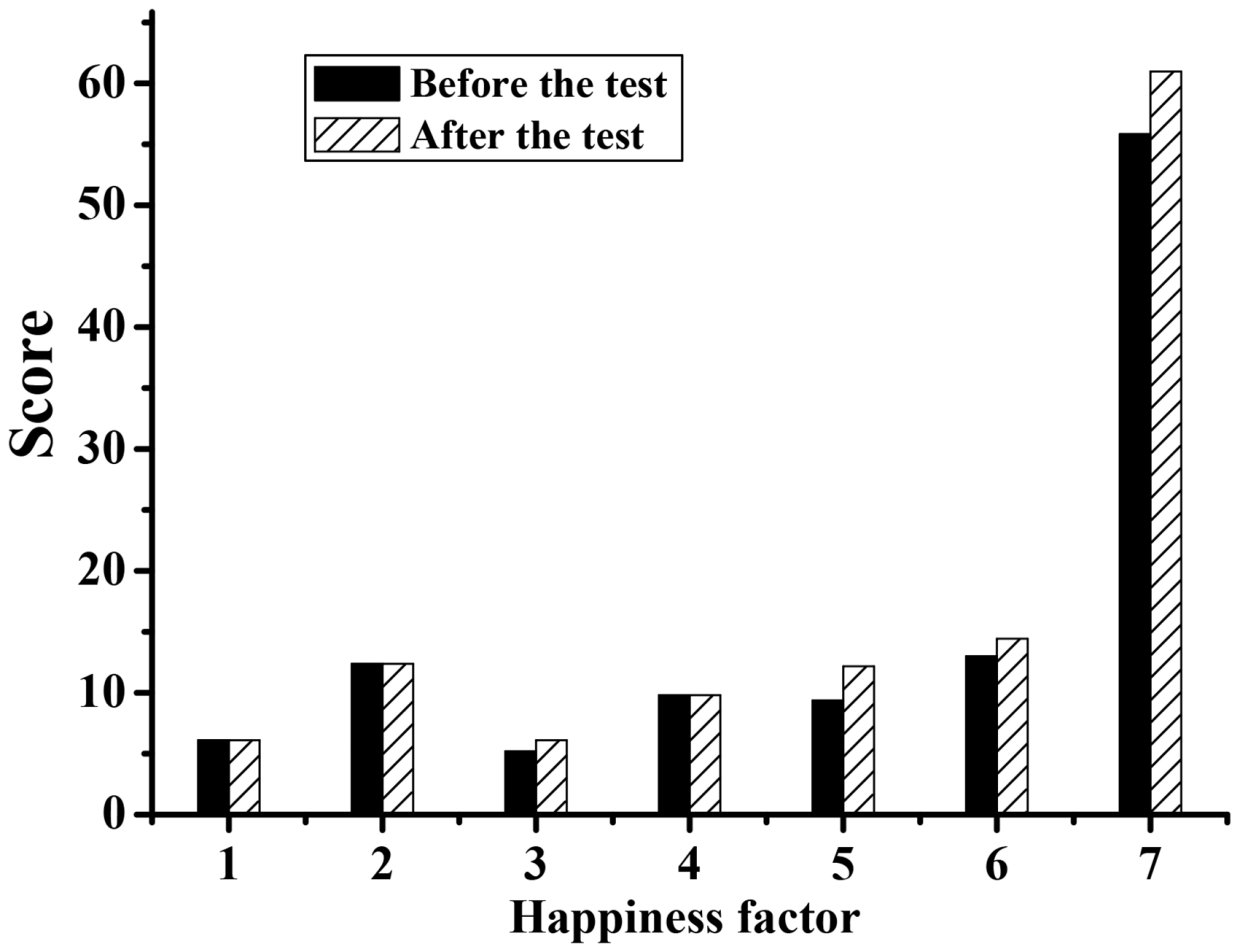
Comparison of the scores of the silver-haired happiness scale in the control group before and after the experiment. (1: worry about health; 2: energy; 3: satisfaction and interest in life; 4: depressed or happy mood; 5: control of emotion and behavior; 6: anxiety; 7: total score).

Figures 5, 6 show the well-being scale of the experimental group and the control group before and after the experiment, respectively. By comparing the data before and after the experiment, it can be concluded that the happiness factors of the experimental group after strengthening the exercise of national music square dance were all higher than those before the experiment. Moreover, the statistical *p*-values of all factors of happiness were all less than 0.05, showing a significant difference, indicating that the happiness of the silver-haired people increased after the exercise of the national music square dance. Among the happiness factors of the silver-haired people in the control group, the three factors of satisfaction and interest in life, control of emotion and behavior, and anxiety were higher than before the experiment, and the statistical *p*-values of the six happiness factors were all greater than 0.05, showing no significant difference, indicating that the happiness of the control group did not change significantly before and after the experiment.

In conclusion, according to the results of the questionnaire survey, the levels of various factors of body self-esteem of the gray-haired people who participated in square dancing were higher than those of the gray-haired people who participated in other sports and non-sports, and there was a significant difference. The subjects in the experimental group had a significant increase in their body self-esteem after 2 months of training in the national music square dance, which showed that square dancing can improve the physical condition of silver-haired people, especially the national music. In terms of happiness improvement, people who participated in square dancing were generally happier than those who did not, and there was a significant difference between them. After 2 months of training in national music square dance, all factors of happiness in the experimental group were higher than those in the control group, and there were obvious differences. The national music square could significantly improve the happiness of the silver-haired people.

Proper physical exercise can improve an individual’s physical quality to a certain extent. The higher the physical quality is, the richer the individual’s positive emotions are and the stronger the individual’s happiness is. Happiness is positively correlated with the level of body self-esteem (Mcgovern et al., 2017; Sutcliffe et al., 2018). Therefore, according to the above experimental results, it can be concluded that the national music square dance plays a positive role in promoting the long-term care and happiness of silver-haired people.

## Conclusion

Square dance is combined with folk music to explore their influence on the improvement of the silver-haired patriarch’s long-term photos and happiness level by means of a literature review, questionnaire survey, and experiments. The results show that ethnic music has a positive impact on the long-term care and happiness of silver-haired people and can improve the quality of life of silver-haired people. However, there are still some deficiencies in this work. This work only discusses the influence of folk music square dance on the long photo and happiness of the silver-haired patriarch from the theoretical perspective and does not further analyze the related factors of the silver-haired patriarch’s photo and happiness under the stimulation of the folk music square. Future work will further explore the factors related to the senior photos and happiness of the silver-haired patriarchs under ethnic music activities based on this work to provide a more comprehensive reference for the intervention of the silver-haired people’s happiness in life. In conclusion, this work provides a theoretical basis for the improvement of subjective well-being among middle-aged and elderly people.

## Data availability statement

The raw data supporting the conclusions of this article will be made available by the authors, without undue reservation.

## Ethics statement

The studies involving human participants were reviewed and approved by ABA Normal University Ethics Committee. The patients/participants provided their written informed consent to participate in this study.

## Author contributions

All authors listed have made a substantial, direct, and intellectual contribution to the work and approved it for publication.

## Funding

This work was supported by following funding: the stage results of the 2020 Ministry of Education Humanities and Social Science Research Planning Fund project “Rescue and Collation and Research on the Folk Music and Old Art Narrative Data of the “Tibetan-Qiang-Yi Corridor” (Project No. 20YJA760111), stage results of the general project of the Southwest Music Research Center in 2018 (project number: xnyy2018008), and the stage results of the general project of Sichuan Ethnic and Folk Music and Dance Research Center in 2019 (project number: MYYB2019-14).

## Conflict of interest

The authors declare that the research was conducted in the absence of any commercial or financial relationships that could be *construed* as a potential conflict of interest.

## Publisher’s note

All claims expressed in this article are solely those of the authors and do not necessarily represent those of their affiliated organizations, or those of the publisher, the editors and the reviewers. Any product that may be evaluated in this article, or claim that may be made by its manufacturer, is not guaranteed or endorsed by the publisher.
